# The Element of Causation in Abdominal Contusions*Read at the annual meeting of the New York State Medical Association, October 12, 1894.

**Published:** 1895-01

**Authors:** Thomas H. Manley

**Affiliations:** New York


### THE ELEMENT OF CAUSATION IN ABDOMINAL CONTUSIONS.*

BY THOMAS H. MANLEY, M. D., NEW YORK.

There exists a considerable discrepancy of opinion on the question of causation in abdominal bruises; or perhaps, it might be more correct to say, on the precise manner in which disorganization succeeds the application of force.

In order to elucidate this subject, several observations have been made on the bodies of those who have succumbed to traumatisms, and on those who have sustained injury, but have survived, the latter presenting such a series of symptoms of local disorders and structural disorganization, as in a certain degree at least, indicated the physical influences brought into operation.

An effort has been made, too, by experimentation on the living dog, to throw light on the subject, by subjecting him to various degrees of violence, and then, after varying intervals, to kill him, and in dissection note the succeeding pathological changes.

With this purpose in view, Dr. B. F. Curtis has conducted an extensive series of interesting experiments on forty-four dogs, supplemented by others in six cadavers (*American Journal of the Medical Sciences*, October, 1887). The abdomens were traumatized by allowing pieces of wood and masses of iron to fall on them. M. Giornadi in the spring of 1879, in Genoa, instituted a very extensive series of experiments in the lower animal, with a similar view.

He applied nearly every conceivable description of force, the animals being in various conditions and placed in various attitudes. His observations were conducted with rare skill and his conclusions are highly interesting. He left little undone, as far as experimentation went. (*Effets des coups-violents sur l'abdomen. Gaz. des hospitaux*, May, 1879.)

Experimentation on the lower animal undoubtedly is not without value in the domain of surgery, but to accept the deductions from it alone, and apply them to the human being without reservation, is a great mistake and calculated to work

*Read at the annual meeting of the New York State Medical Association, October 12, 1894.

much evil. In this particular class of cases the analogy or comparison is not close, and conclusions reached will accordingly have but a very limited range of application when extended to practical surgery.

The greater number of contusions, or bruises on the abdomen which we encounter on the human subject, it is quite impossible to induce at all on the lower animal; *e.g.*, as falls from great heights, machinery, railroad accidents, run-over injuries and the like. The law, public sentiment and humane feeling alike demand that the animal must be anæsthetized, whereby he is unable to avail himself of those auxiliary safeguards with which he is provided, in the event of sudden accident. The muscles are relaxed and the relation of the organs are in consequence materially altered. Besides all this there is little or no analogy in structural composition and arrangement.

The quadruped moves in a horizontal position of the body, while the attitude of man is perpendicular. The entrails of a dog are very thick and tough, and tolerate in a most remarkable degree any description of manipulation regardless of prophylaxis against sepsis. His recuperative powers after trauma are marvelous. His intestine may be removed by the foot or yard, the ends sewed together, and he will scarcely miss a meal, so slight is the constitutional disturbance. His peritoneum, which is nearly as tolerant of handling as his integument, is quite immune against trauma.

But, with man, he receives the main impact of force, in full consciousness, and though his peritoneal cavity is the most exposed to percussive in crushing force, it often escapes. Nature has made such provision for its protection through sudden changes of attitude, muscular contraction and the defenses provided by the extremities that serious damage of it is much less frequent than of the apparent better protected cranial or thoracic cavities. In a *second's warning* the muscular girth of the belly sends the floating viscera up behind the shelving vault of the lower thorax, the chest is inclined and the knees drawn up. Indeed, as a matter of fact, serious non-penetrating injuries are seldom seen, except when the body is suddenly caught and fixed, or one is hit when off his guard.

The human intestine is very thin and fragile as compared with the dog, and its serous-membrane is so exquisitely sensitive and intolerant, that it never can be exposed without danger to life. It is therefore evident that in order to pursue an intelligent and practical study of this class of lesions, our observations must be made rather in the wards of the hospital and the autopsy room, than in a laboratory.

### MORTAL CONCUSSION OF THE ABDOMEN BY BLOWS OVER THE EPIGASTRIUM.

A few cases are on record of instant death following blows over the epigastrium.

Ashhurst declared it as his opinion "that death may follow a severe blow over the abdomen." (*Ashhurst's Surgery*, p. 391.)

Mr. Thomas Bryant is unequivocal on this point, and says that "under certain circumstances, a trifling blow may give rise to alarming symptoms." (Bryant's *System of Surgery*, p. 217.) Agnew was reserved on this question. Pirrie believed that blows over the stomach or duodenum were more dangerous than on other parts of the abdomen. (*Principles of Surgery*, p. 595.) Le Gros Clark said that "if the doctrine be true that a blow over the abdomen may cause death, it must be a very rare accident." (*Encyclopædia of Surgery*, p. 988.) Holmes practically repudiated the doctrine. Guthrie believed that violent blows over the abdomen might lead to ultimate absorption of muscle and gradual relaxation, favoring hernia. (Guthrie *Surg. Obs.*, p. 312.) Gaut adds his testimony to the probability of blows over the abdomen producing fatal results. (*Science nd Princip]es of Surgery*, vol. i., p. 532.)

With a view of determining as fully as possible the recent views of surgeons on abdominal contusions, the current home and foreign literature issued in the past fifteen years was examined, since the time that laparotomy became a recognized surgical procedure. One of the most conspicuous features of the search was the comparative scarcity of abdominal contusions chronicled during that period, which would imply that this subject has not received the attention which its importance merits.

During that period I could find no case on record of sudden death from a blow over the abdomen without well-marked pathological alterations succeeding.

Modern observation and experimentation all tend to discount and repudiate the assumption that death is commonly possible by a blow anywhere over the abdominal walls in one of sound health, without structural lesion following.

Mortal concussion of the brain and spine are to-day denied by some of our noted authors, and probably mortal concussion of the abdomen, without central pathological changes has little more to support and perpetuate it than the parrot-like practice in vogue in the past of servilely quoting from one work to another, notions and opinions which were wanting the stamp of personal observation and had nothing to support them except their hoary antiquity.

Sudden, concentrated violence applied to the abdomen undoubtedly may cause almost instant death, but not with the frequency heretofore supposed. But cases of sudden death are on record from blows over the cranium, the neck or the thorax.

A person is suddenly hit over the abdomen in full inspiration when the lower lobe of the left lung has descended behind the diaphragm, he gasps for a moment, shock is communicated through the reflexes to the respiratory center and for an instant he is unable to breathe.

A similar degree of violence applied over the trachea, the thorax or face may produce a somewhat similar train of symptoms. The proximity of the solar plexus, the præcordia and intimate relations of the terminal filaments of the pneumogastric and sympathetic ganglia, have been supposed to render the epigastric region more susceptible to injury than others. There are, however, but very few well-authenticated cases on record of immediate death from blows over the abdomen. We never see them occur as a result of crushes. Some authorities deny that a blow can produce death unless the individual is suffering from organic disease, is greatly exhausted or fatigued, as a prize-fighter toward the close of the contest. No surgeon has ever been an eye-witness to such an accident; therefore, it is probable that these cases on record were complicated by concomitant pathological conditions, and with them the blow over the stomach is only the fatal climax. Probably in most instances, the patient, in the midst of great excitement, when struck, psychic influences play a dominant part in paralyzing the heart's action.

CAUSATION IN CASES OF LACERATION OR RUPTURE OF THE ABDOMINAL VISCERA SUCCEEDING PERCUSSIVE OR CRUSHING VIOLENCE.

The abdominal viscera, in the performance of their functions, do so in obedience to physical and vital laws in connection with pneumatics, hydraulics and motion. The integrity of the viscera is preserved by the harmonious action of these natural forces. A practical knowledge of these is quite indispensable for an intelligent comprehension of the mechanical element in all instances of serious, non-penetrating abdominal injuries. It goes without saying that a familiarity with the structural anatomy and pathological laws in operation here after injury is presupposed.

In the present instance, attention will be chiefly directed to the mechanics of the subject as an element in ætiology.

For our present purpose we may regard the abdomen as a sac with two openings, one above and one below. Within it are the solid organs, a tubular structure, containing gases, fluids and solids, besides the great blood trunks and other accessory structures.

Its contents are tethered to the spinal column and diaphragm and are in incessant motion.

Its vulnerable areas are, the anterior, lateral and posterior. The latter is fixed solid, and resistant, so that violence, when coming through the lumbar, must first spend its energy on the spine.

As a general rule, in abdominal bruises the force comes from before.

This force is of two qualities:

First, percussive or contusive, and, secondly, crushing.

In the majority of cases, the viscera are disorganized by being directly crushed against the bodies of the spinal-column. In the minority, trauma is probably sustained through percussive force as by blows, kicks or falls.

(TO BE CONTINUED.)

---

FOR INTERCOSTAL NEURALGIA.—

℞. Linimenti belladonnæ ................ f. ℥j.

Linimenti chloroformi ................ f. ʒiv.

Linimenti opii ................ ad. f. ℥ii.

Misce et fiat linimentum.

To be well rubbed over the painful area.—*The Practitioner.*

